# Clinic Reports

**Published:** 1881-04

**Authors:** 


### CLINIC REPORTS.

DECEMBER 11th, 1880.

MALIGNANT TUMOR—EPITHILOMA.—Young man, aged 22. Four years ago this patient had an epithiloma removed from his lower lip, also, a tumor moved from below and to outside of left eye. His grandfather died from cancer. He appeared at the clinic with a tumor over the body of right side of lower jaw, dipping down in neck, which was slightly movable. It was probably a return of the epithiloma. It was malignant in character, and a remarkable and serious case. Dr. Maclean said: "I never saw a case of epithiloma in so young a person, and it is a very unusual thing." Patient being chloroformed, a nearly perpendicular incision was made, about three and a half inches in length, commencing about an inch below the lobe of the right ear and extending downward. The tumor was then dissected out, leaving most of the body of jaw bare; though the disease did not seem to have affected the bone. A few vessels were tied, and there was but little blood lost. The parts were stitched together with horse hair ligatures; they being the best where they will suffice, and are strong enough. The submaxillary gland was removed with the tumor, which was adherent to it.

DECEMBER 15th.—Patient doing well. The wound was not entirely healed, but there was no discharge, except blood.

JANUARY 5th, 1881.

EPITHILOMA.—This patient, a man, aged 62 years, came before the class with a form of epithiloma of the tongue. There was no glandular enlargment in the neck, and the case a favorable one for operation. The patient used to smoke, but stopped that two years ago. In October last, he tasted some unknown root that he found in the woods, and a smarting sensation and a slight protusion on the tongue followed. The patient being chloroformed, a gag was put between his teeth to hold his mouth open. His tongue was held out with forceps, all the disease cut away, and actual cautery applied to destroy any possible remaining traces of the disease, and to stop the hemorrhage. Cancer of the tongue is very dangerous, and the operation of excising the whole tongue is practiced. In 1862, Prof. Syme performed the third, and first successful operation of excising the whole tongue.

JANUARY 8th, 1881.

EPITHILOMA.—In this patient the epithiloma was on the upper lip and extending to the alveolar process. The patient was a man aged 51. Chloroform was given him, and a portion of the lip and process were removed. He had been operated on before, and the disease returned; but no bone was removed at the first operation.

JANUARY 8th, 1881.

NAEVUS BY ANASTOMOSIS.—The patient was a boy aged 9 years. The disease was in the upper lip, and the nævus was very large. The boy was chloroformed, a clamp put on the lip on each side, and both poles of an electric battery ending in needles were run into the growth. This had the effect to somewhat lessen the nævus. January 22nd actual cautery was resorted to, and a portion of the growth removed. Dr. Maclean said he had two very satisfactory results from the use of the battery. Persulphate of iron in strong solution injected into a nævus will often prove valuable.

